# Heart Failure Solutions: The PeriCut Device as a Treatment for HFpEF

**DOI:** 10.1016/j.jacbts.2024.02.010

**Published:** 2024-04-22

**Authors:** Barry A. Borlaug, Mark Strong

**Affiliations:** a Mayo Clinic, Rochester, Minnesota, USA; b Heart Failure Solutions, Shoreview, Minnesota, USA

Morbidity and mortality are increased in heart failure with preserved ejection fraction (HFpEF) patients because of elevated pulmonary capillary wedge pressures (PCWP). It is well-established that pericardial constraint increases external pressure on the heart and amplifies the increase in PCWP.^1^ In patients with HFpEF, a substantial component of pressure elevation in the ventricle is related to external contact pressure exerted by the intact pericardium.

**Figure 1:**
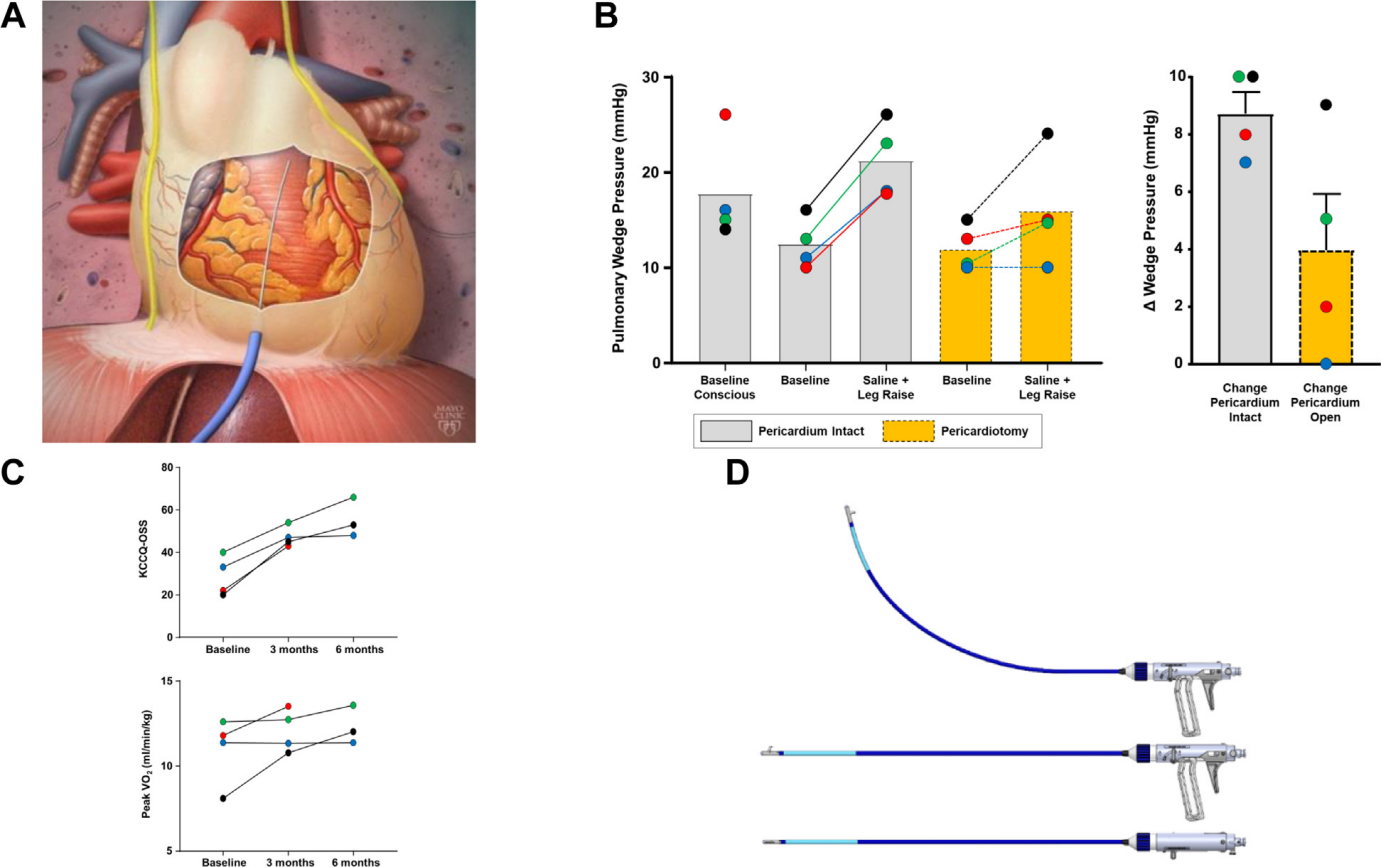
Figure Panels (A) Schematic illustration of the approach for subxiphoid pericardiotomy is shown. Favorable blunting of the increase in pulmonary capillary wedge pressure with volume loading was observed following pericardiotomy in a series of 4 patients with heart failure with preserved ejection fraction (B), in whom there were also trends for improvement in Kansas City Cardiomyopathy Questionnaire Overall Summary Score (KCCQ-OSS) and peak oxygen consumption during exercise (Vo_2_) (C). (D) The final PeriCut device prototype is shown.

The idea of reducing pericardial restraint in patients with HFpEF through pericardiotomy has recently been explored through a series of studies (Figure 1).^2^, ^3^, ^4^, ^5^ In acute and chronic animal preparations (canine and swine), the increase in left ventricular filling pressures with volume loading was favorably reduced after opening the pericardium, an effect that did not require a full pericardiectomy, and this salutary effect was sustained out to 4 weeks following the pericardiotomy procedure in an animal model of HFpEF.^2^^,^^4^

Following the success of the animal studies, an acute pilot study was conducted in patients undergoing clinically indicated cardiac surgery.^3^ Participants underwent assessment of PCWP at baseline and following acute volume loading with the pericardium intact. The pericardium was opened for surgical access, and the hemodynamic measurements were repeated at baseline and during the same preload challenge. As in the animal experiments, there was no significant effect on PCWP measured at baseline, but the increase in PCWP during the volume loading maneuver was reduced by 67%, an effect that would be expected to lead to favorable hemodynamic effects during other stresses associated with increased venous return, such as physical exertion.

Of the 19 participants enrolled in this pilot study, 10 displayed pathophysiologic findings suggestive of underlying HFpEF, including dyspnea with abnormal E/eʹ and/or elevated N-terminal pro–B-type natriuretic peptide (NT-proBNP).^3^ After pericardiotomy, theses 10 participants with features of HFpEF displayed a greater (more favorable) attenuation of the increase in PCWP with volume loading (−7 ± 2 mm Hg vs −3 ± 2 mm Hg; *P* = 0.003).

The first-in-patient pilot study was performed testing the effects of surgical pericardiotomy on 4 women with HFpEF and NYHA functional class III symptoms (age 75 ± 4 years, body mass index 34.8 ± 4.5 kg/m^2^, EF 60% ± 5%, E/eʹ 17 ± 12, peak Vo_2_ 11.0 ± 2.0 mL/kg/min, Kansas City Cardiomyopathy Questionnaire [KCCQ] score 29 ± 9).^5^ Access to the pericardial space was obtained using off-the-shelf surgical tools through a left minithoracotomy. As in the prior studies, PCWP was measured at baseline and with volume loading with pericardium intact. A 4-cm anterolateral pericardiotomy was then performed surgically under direct thoracoscopic visualization, parallel to the left phrenic nerve using low-voltage electrocautery and off-the-shelf surgical tools. Before the procedure, after induction of general anesthesia, and immediately postpericardiotomy, hemodynamic measurements were obtained without and with volume loading and leg elevation. Subjects then returned for clinical assessments at 3 and 6 months postprocedure.

The procedure was successfully completed without complication in all subjects.^5^ With repeat volume loading and leg elevation, the pulmonary artery wedge pressure was significantly lower than preprocedure (16 ± 6 mm Hg vs 21 ± 4 mm Hg; *P* = 0.046 by *t*-test) with the pericardium intact (Figure 1). Patients underwent repeat cardiopulmonary exercise testing and cardiac magnetic resonance imaging at follow-up visits, in addition to the KCCQ. There were numerical increases in the KCCQ and peak Vo_2_, although not anticipated to be statistically significant due to the small sample size. One serious adverse event occurred that was determined to be related to the study intervention; the first patient enrolled developed pericarditis and inflammatory pericardial effusion without tamponade on postoperative day 14 after discharge from the hospital, which was successfully treated with pericardiocentesis. Following review with the trial data safety monitoring board, the decision was made to modify the protocol to administer colchicine proactively before surgery in all remaining patients to decrease the risk of inflammatory postpericardiotomy syndrome, and none of the remaining patients experienced this adverse event.

Three of the 4 patients were able to complete the final 6-month visit (1 patient could not return due to the COVID-19 pandemic; however, the patient was contacted by phone and was alive and well).^5^ Repeat echocardiography and cardiac magnetic resonance imaging showed no changes in left ventricular function or chamber dilation that would be suggestive of an adverse response. Given the patient-reported improvement of symptoms and activity tolerance, along with favorable hemodynamic effects and objective improvements in exercise, it is hypothesized that minimally invasive pericardiotomy might be helpful to abrogate the increase in filling pressures that develops during activities of daily living in patients with HFpEF, and in doing so, improve health status, reduce symptom severity, and improve exercise function. However, the risks and recovery with open sternotomy or minithoracotomy are factors limiting application of this treatment approach.

The newly designed PeriCut System consists of a steerable catheter, a sheath, and a dilator. The 18-F catheter contains many features to safely perform the pericardiotomy percutaneously through a minimally invasive, subxiphoid approach (Figure 1). The catheter handle has a rotating knob used to maneuver and deflect the catheter shaft to ensure proper placement. The handle incorporates a trigger that deploys and retracts a surgical blade capable of performing the pericardial cut. In addition, the catheter includes dipole pacing electrodes on the distal tip and proximal housing, assisting with keeping the catheter safely away from the phrenic nerves. The blade includes a port that allows the physician to inject contrast solution to visualize the window of the completed cut.

The PeriCut catheter-based investigational device is designed to perform a pericardiotomy through minimally invasive access to the pericardial space to treat patients with HFpEF, in addition to guideline-directed medical therapy. The hypothesized benefit to the patient will be complete or partial reductions in symptoms of HFpEF, including dyspnea, that are related to high left ventricular filling pressures. This could result in reduced symptom severity, improved health status and exercise function, and reduced need for hospitalizations due to heart failure. Chronic reduction in filling pressures may also reduce pulmonary artery pressures and decrease the incidence of right ventricular dysfunction, which is associated with increased mortality. Therefore, this therapy could potentially improve HFpEF patient survival.

In summary, PeriCut may favorably transform the heart failure practice by providing a new treatment option for this underserved patient population. A first-in-patient trial is expected to begin enrollment.
